# Impact of retinoic acid exposure on midfacial shape variation and manifestation of holoprosencephaly in *Twsg1* mutant mice

**DOI:** 10.1242/dmm.018275

**Published:** 2014-12-02

**Authors:** Charles J. Billington, Brian Schmidt, Ralph S. Marcucio, Benedikt Hallgrimsson, Rajaram Gopalakrishnan, Anna Petryk

**Affiliations:** 1 Department of Pediatrics, University of Minnesota, Minneapolis, MN 55454, USA.; 2 Department of Genetics, Cell Biology and Development, University of Minnesota, Minneapolis, MN 55454, USA.; 3 Department of Orthopedic Surgery, University of California, San Francisco, CA 94110, USA.; 4 Department of Cell Biology & Anatomy, University of Calgary, Calgary, AB T2N 4N1, Canada.; 5 Diagnostic/Biological Sciences, School of Dentistry, University of Minnesota, Minneapolis, MN 55455, USA.

**Keywords:** Twisted gastrulation, *Twsg1*, Bone morphogenetic protein, Holoprosencephaly, Retinoic acid, Apoptosis, Oxidative stress

## Abstract

Holoprosencephaly (HPE) is a developmental anomaly characterized by inadequate or absent midline division of the embryonic forebrain and midline facial defects. It is believed that interactions between genes and the environment play a role in the widely variable penetrance and expressivity of HPE, although direct investigation of such effects has been limited. The goal of this study was to examine whether mice carrying a mutation in a gene encoding the bone morphogenetic protein (BMP) antagonist twisted gastrulation (*Twsg1*), which is associated with a low penetrance of HPE, are sensitized to retinoic acid (RA) teratogenesis. Pregnant *Twsg1^+/−^* dams were treated by gavage with a low dose of all-trans RA (3.75 mg/kg of body weight). Embryos were analyzed between embryonic day (E)9.5 and E11.5 by microscopy and geometric morphometric analysis by micro-computed tomography. P19 embryonal carcinoma cells were used to examine potential mechanisms mediating the combined effects of increased BMP and retinoid signaling. Although only 7% of wild-type embryos exposed to RA showed overt HPE or neural tube defects (NTDs), 100% of *Twsg1^−/−^* mutants exposed to RA manifested severe HPE compared to 17% without RA. Remarkably, up to 30% of *Twsg1^+/−^* mutants also showed HPE (23%) or NTDs (7%). The majority of shape variation among *Twsg1^+/−^* mutants was associated with narrowing of the midface. In P19 cells, RA induced the expression of *Bmp2*, acted in concert with BMP2 to increase p53 expression, caspase activation and oxidative stress. This study provides direct evidence for modifying effects of the environment in a genetic mouse model carrying a predisposing mutation for HPE in the *Twsg1* gene. Further study of the mechanisms underlying these gene-environment interactions *in vivo* will contribute to better understanding of the pathogenesis of birth defects and present an opportunity to explore potential preventive interventions.

## INTRODUCTION

Holoprosencephaly (HPE) is a malformation characterized by inadequate or absent midline division of the embryonic forebrain. Incomplete brain septation is accompanied by corresponding midline facial defects in ~80% of cases (Geng and Oliver, 2009) and, less frequently, jaw defects (Pauli et al., 1983). HPE is the most common defect of the developing forebrain with an incidence of 1 in 250 conceptuses and about 1 in every 10,000 at term (Orioli and Castilla, 2010; Roessler et al., 1996). An important feature of HPE is its incomplete penetrance and expressivity. Even in families with defined mutations, some individuals can have no recognizable defects, some have mild forms (referred to as microforms, such as hypotelorism, midfacial hypoplasia, or a single maxillary central incisor) and some are severely affected with cyclopia or proboscis (Roessler et al., 1996). The basis of this phenotypic variability is poorly understood.

HPE can result from widely diverse causes, including both genetic and environmental etiologies. It has been speculated that genetic and environmental factors can have a cumulative effect, accounting for its varied penetrance and expressivity (Ming and Muenke, 2002). The most common genetic cause of HPE in humans is mutations in *SHH* (Roessler et al., 1996). Some examples of environmental factors that have been associated with development of HPE in humans are ethyl alcohol, poorly controlled maternal diabetes mellitus, retinoic acid (RA) (Cohen and Shiota, 2002) and hypoxia-ischemia (Siebert, 2007). All of these environmental factors are associated with elevated levels of reactive oxygen species (ROS) (Aoto et al., 2008; Davis et al., 1990; Kay et al., 2000; Ornoy, 2007), suggesting that oxidative stress has a role in mediating their teratogenic effects.

Experimental models of HPE in which to study these interactions are very limited because unlike humans, mice carrying classical HPE gene mutations do not usually show phenotypic variability. For example, disruption of the SHH pathway in mice has profound effects on embryonic development with all *Shh*-null embryos manifesting severe HPE (Chiang et al., 1996), whereas in humans only 37% of carriers of *SHH* mutations develop HPE (Cohen, 1989). Other, less classical mouse models of HPE, however, do show incomplete penetrance and phenotypic variability, making them potentially more amenable to environmental manipulation with a resultant shift in a phenotypic outcome. For example, loss of bone morphogenetic protein (BMP) antagonists, such as chordin, noggin or twisted gastrulation (TWSG1), leads to a reduction in *Shh* expression in the ventral neural midline and recapitulates a spectrum of HPE phenotypes in mice (Anderson et al., 2002; Lana-Elola et al., 2011; Petryk et al., 2004). As with BMPs, exogenous RA can also lead to loss of *Shh* expression and HPE (Helms et al., 1997; Sulik et al., 1995). Although it is currently unknown whether mice with disrupted BMP signaling are more susceptible to RA teratogenic effects, there is evidence that both pathways can cooperate during development, for example, during vertebrate limb outgrowth, by inducing interdigital apoptosis (Rodriguez-Leon et al., 1999).

**TRANSLATIONAL IMPACT**

**Clinical issue**

Holoprosencephaly (HPE) is the most common defect of the developing forebrain and has an incidence of 1 in 250 conceptuses and about 1 in every 10,000 at term. It is characterized by inadequate or absent midline division of the embryonic forebrain and midline facial defects. A perplexing feature of HPE, as well as of other craniofacial syndromes, in humans is their widely variable penetrance and expressivity even in the case of the same single gene mutation within the same family, with some individuals having severe defects, some mild defects and some being unaffected. It is currently unknown what causes manifestation of HPE in genetically at risk individuals, but it has been speculated that environmental factors might play a role. This work investigates the effects of environmental exposure to teratogens in a mouse model predisposed to HPE.

**Results**

Twisted gastrulation (*Twsg1*) mutant mice serve as a model of human HPE because they show incomplete penetrance and a range of defects among homozygotes. In this study, the authors demonstrated that *Twsg1* mutants show increased susceptibility to the teratogenic effects of relatively low doses of retinoic acid (RA) that in control mice cause few, if any defects. The exposure to RA was performed at embryonic day 7.5, which is the most sensitive window for teratogen-induced HPE (corresponding to the 3rd to 4th week post-fertilization in humans). Remarkably, even *Twsg1* haploinsufficiency exacerbated teratogenic effects of prenatal RA exposure. The majority of midfacial shape variation among *Twsg1^+/^*^−^ mutants was associated with narrowing of the midface, as demonstrated by micro-computer tomography (microCT) analysis. Given that the only known action of TWSG1 is through binding bone morphogenetic proteins (BMPs) in the extracellular space, the authors hypothesized that BMP-RA interactions could contribute to the enhanced expressivity of HPE in this mouse model. To test this, P19 embryonic carcinoma cells were used as an *in vitro* model to elucidate the mechanisms mediating these gene-environment interactions. In P19 cells, RA induced the expression of *Bmp2* and its downstream targets *Msx1* and *Msx2*, and acted in concert with BMP2 to increase apoptosis, p53 target gene expression and oxidative stress, suggesting a role for these pathways in inducing cell death and exacerbating the disease outcome.

**Implications and future directions**

This study provides direct evidence of the effects of the environmental exposure to teratogens on craniofacial development in a genetic mouse model carrying a predisposing mutation for HPE. Further study of the mechanisms underlying these gene-environment interactions *in vivo* will contribute to better understanding of the pathogenesis of birth defects and will represent an opportunity to explore potential preventive interventions.

The primary goals of this work were (1) to examine whether a mutation in a gene encoding the BMP-binding protein TWSG1 confers susceptibility to RA exposure, and (2) whether this effect can be quantified by micro-computed tomography (microCT) of the craniofacial region. We chose the *Twsg1* mutant mouse model because it has a relatively low baseline incidence of HPE and because the craniofacial defects in these mice are caused by an increase in apoptosis (MacKenzie et al., 2009). A secondary goal was to examine the potential underlying mechanisms of HPE, using P19 cells as a validated *in vitro* model of BMP-RA interactions. We hypothesized that *Twsg1^−/−^* mice would be particularly sensitive to the subteratogenic effects of RA, the midface would be most significantly affected, and the effects of a combined treatment of P19 cells with BMP and RA would be mediated through upregulation of apoptosis.

## RESULTS

### *Twsg1*^−/−^ mice are sensitized to retinoic acid teratogenesis

Our first step in examining the sensitivity of TWSG1-deficient mice to RA was to establish an appropriate treatment dosage that would cause a low but observable incidence of defects in wild-type (WT) mice, which could then be used to assess the sensitivity of TWSG1-deficient mice. Given that a dose of all-trans RA (ATRA) of 7.5 mg/kg of body weight has been previously shown to cause significant HPE (Kotch et al., 1995), we also tested a lower dose of 3.75 mg/kg. Treatment with 7.5 mg/kg of ATRA was overwhelmingly teratogenic and led to 94% of WT embryos showing defects with about two thirds of the embryos showing HPE and one third with a neural tube defect (NTD) (Table 1). However, treatment with 3.75 mg/kg of body weight of ATRA led to 7% of embryos being affected, with a vast majority of these affected embryos showing HPE (Table 1). Therefore, we selected 3.75 mg/kg of ATRA (referred to as low dose ATRA) as our dose for future experiments with *Twsg1*^+/−^ and *Twsg1*^−/−^ mice.

**Table 1.**
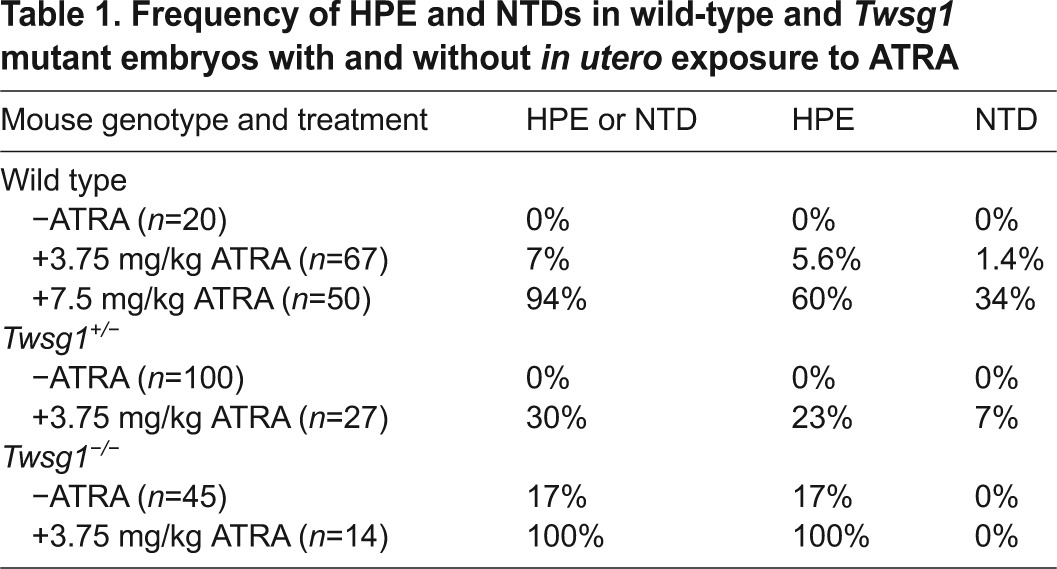
Frequency of HPE and NTDs in wild-type and *Twsg1* mutant embryos with and without *in utero* exposure to ATRA

**Fig. 1.**
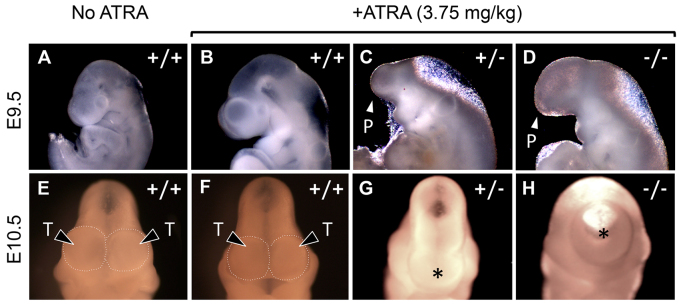
**Phenotypic analysis of *Twsg1* mutant embryos exposed *in utero* to a low dose (3.75 mg/kg of body weight) of ATRA.** (A–D) Lateral views of E9.5 embryos. (A) WT untreated embryo; (B) normal appearance of ATRA treated WT embryo; (C,D) *Twsg1* mutant embryos treated with ATRA showing proboscis (P) and absence of telencephalic vesicles. (E-H) Frontal views of E10.5 embryos. (E) WT untreated embryo; (F) normal telencephalic vesicles (T; outlined by a dotted line) of an ATRA-treated WT embryo; (G,H) HPE (marked by *) in *Twsg1* embryos treated with ATRA.

Although only 7% of WT embryos exposed to low dose ATRA showed overt HPE or NTDs, 100% of *Twsg1*^−/−^ mutants manifested HPE compared to 17% without exposure to ATRA (Table 1; Fig. 1; *P*=6×10^−12^). This rate of defects is far more than what would be expected based solely on adding the prevalence of defects from this dose in WT mice and untreated *Twsg1^−/−^* mice. Remarkably, even 30% of heterozygous *Twsg1* mutants, which are phenotypically normal without ATRA exposure, showed neural defects (predominantly HPE). This represents a statistically significant increase over the incidence seen in WT embryos with the same dosage (*P*=0.01) and likewise over the 0% incidence seen in untreated heterozygotes (*P*=1×10^−6^). Thus, TWSG1 deficiency increased the teratogenic effect of low dose ATRA in both the homozygous and heterozygous states.

### Geometric morphometric analysis of the facial shape of *Twsg1*^+/−^ mice demonstrates a continuum of midfacial dysmorphology after exposure to a low dose of ATRA

**Fig. 2.**
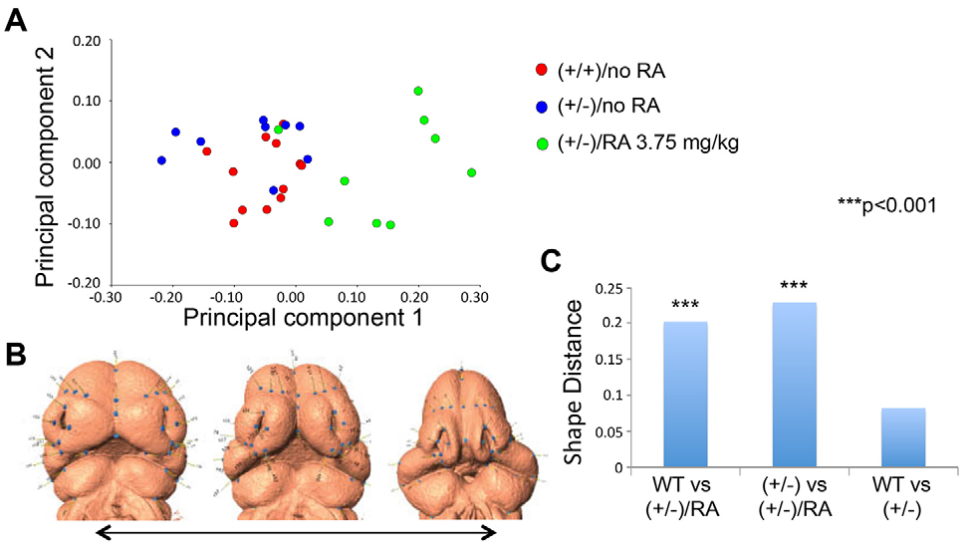
**GM analysis of facial shape.** (A) Principal component analysis (PCA) based on a GM analysis of facial shape in WT and *Twsg1^+/−^* mouse embryos at E11.5 with or without *in utero* exposure to a low dose of ATRA (RA) at E7.5. PC analysis of landmark data shows that the majority of shape variation is associated with narrowing of the midface and that PC1 discriminates between treatment groups. PC1 distinguishes between treated embryos and both WT and heterozygous *Twsg1* mutant embryos. (B) 3D morphing showing variation along PC1. (C) Procrustes distances between groups. *P*-values were obtained by permutation of the Procrustes distance.

Although *Twsg1^−/−^* embryos exhibited severe HPE phenotypes (cyclopia or proboscis) after *in utero* exposure to a low dose ATRA, *Twsg1^+/−^* showed a range of defects of variable severity. To quantify these defects, geometric morphometric (GM) analysis was employed. The analysis included only *Twsg1^+/−^* embryos because severe HPE phenotypes in homozygotes precluded landmark assignment. Principal component 1 (PC1), which reflects narrowing of the midface, was the only PC that discriminated between treatment groups and accounted for 49% of the total variance. As shown in Fig. 2, whereas untreated *Twsg1^+/−^* embryos clustered with WT embryos, those that were affected by low dose ATRA treatment could be clearly discriminated along PC1. Thus, ATRA treatment resulted in a continuum of midfacial narrowing in mice heterozygous for *Twsg1* mutation.

### ATRA induces the expression of RA-responsive genes in P19 cells

**Fig. 3.**
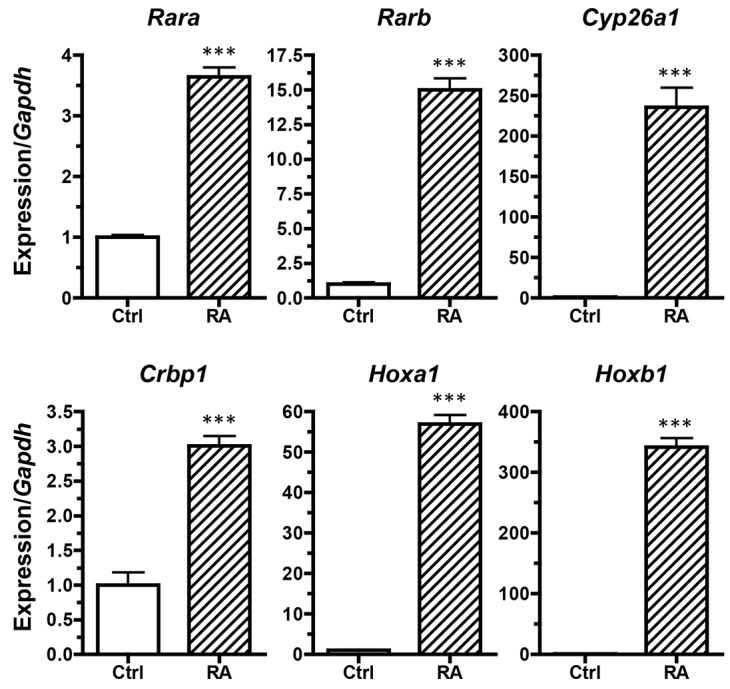
**RA target gene expression in response to ATRA treatment in P19 cells as assessed by qPCR.** The P19 cell cultures were treated with either DMSO vehicle (Ctrl) or 1 μM ATRA (RA) for 24 hours, with transcript levels quantified by qPCR. Gene expression was normalized to the expression of *Gapdh*, and is shown relative to the DMSO-vehicle-treated control expression (set at 1). The induction of RA targets in P19 cells indicates the suitability of the P19 cell model for investigating responses to RA signaling. Results are mean±s.e.m. (*n*=6). ***P<0.001 (Student’s *t*-test).

To test potential mechanisms underlying the acute sensitivity of *Twsg1* mutant mice to ATRA, we selected P19 mouse embryonic carcinoma cells as an experimental system because they resemble embryonic cells, represent a homogenous cell population that is amenable to quantitative assays and have been used by others as a model for BMP-retinoid signaling interactions (Fujita et al., 1999; Glozak and Rogers, 1996; Glozak and Rogers, 1998). P19 cells have been previously reported to be sensitive to retinoids (Xi and Yang, 2008). We were able to confirm this sensitivity by observing the transcriptional induction of several known RA target genes after 1 μM ATRA treatment, including RA receptors α and β (*Rara* and *Rarb*) (Balmer and Blomhoff, 2002; Sucov et al., 1990), the RA hydrolase *Cyp26a1* (Loudig et al., 2000), *Crbp1* (Xu et al., 2001), and the Hox transcription factors *HoxA1* and *HoxB1* (Balmer and Blomhoff, 2002; Dekker et al., 1993; Dupé et al., 1997) (Fig. 3).

### ATRA upregulates the expression of *Bmp2* and its downstream targets in P19 cells

**Fig. 4.**
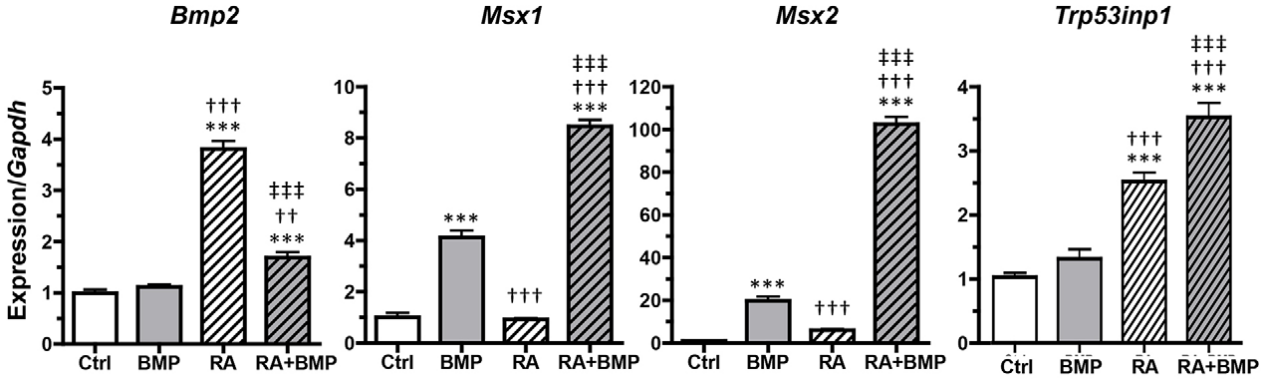
**Gene expression levels in response to exogenous BMP2 and ATRA as assessed by qPCR.** P19 cell cultures were treated for 24 hours with vehicle control (0.00001% BSA, 0.001% DMSO, Ctrl), 10 ng/ml recombinant human BMP2 (BMP2), 1 μM ATRA (RA), or 10 ng/ml rhBMP2 and 1 μM ATRA (RA+BMP). Gene expression was normalized to the expression of *Gapdh*, and is shown relative to the mean vehicle-treated control expression (set at 1), with transcript levels quantified by qPCR. ATRA induced both BMP2 and its downstream targets in P19 cells. Combined BMP and ATRA treatment resulted in a significant increase in *Trp53inp1* expression. Results are mean±s.e.m. (*n*=6). ***P<0.001 compared with Ctrl; ^†††^*P*<0.001 compared with BMP treatment; ^††^*P*<0.01 compared with BMP treatment; ^‡‡‡^*P*<0.001 compared with BMP+ATRA treatment (Tukey’s test).

Given that the only known mode of action for TWSG1 is through regulation of BMP signaling, it was essential that the P19 cells be competent to respond to BMPs to mimic what occurs *in vivo*. Although there is some evidence that TWSG1 can promote BMP activity in some species, in mice it appears to act mostly as a BMP antagonist (Larraín et al., 2001; Nosaka et al., 2003; Oelgeschläger et al., 2003; Petryk et al., 2005; Ross et al., 2001; Sotillo Rodriguez et al., 2009; Wills et al., 2006). We examined the expression of several BMP pathway genes and BMP targets after BMP2 treatment alone, after ATRA treatment alone and after combined treatment (Fig. 4). We found, consistent with previous reports (Heller et al., 1999), that *Bmp2* was upregulated in response to ATRA. The BMP targets *Msx1* and *Msx2* (Davidson, 1995; Liu et al., 2005; Vainio et al., 1993) showed a significant induction upon treatment with BMP2 alone. With ATRA alone, *Msx1* was not induced, whereas *Msx2* was increased 6.2-fold compared to the control group, although not sufficiently to test as statistically significant. However, when BMP2 and ATRA treatments were combined, both *Msx1* and *Msx2* showed dramatically higher induction than with BMP2 alone (~2-fold increase for *Msx1* and ~5-fold increase for *Msx2* compared to BMP2 alone).

### The p53 pathway is activated in P19 cells treated with BMP and RA together

The upregulation of the p53 target *Trp53inp1* (Tomasini et al., 2003) is indicative of increased p53 transcriptional activity in the cell and activation of the p53 pathway. Following treatment with BMP2, the expression of *Trp53inp1* was not significantly changed (Fig. 4). ATRA, however, significantly increased *Trp53inp1* expression. In response to a combined treatment with ATRA and BMP2, significantly more expression was observed beyond even that seen with ATRA alone.

### RA acts in concert with BMP to increase caspase 3 and 7 activation in P19 cells

**Fig. 5.**
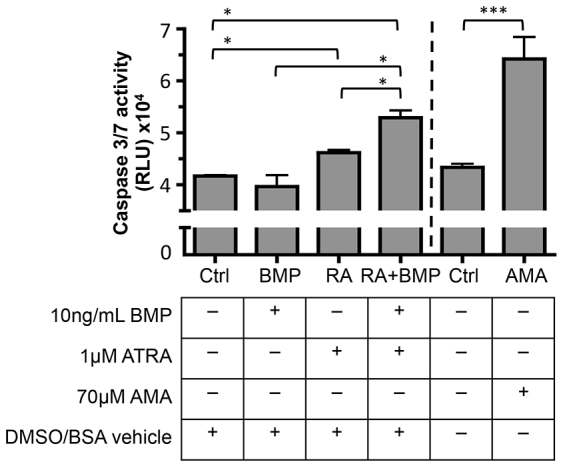
**Caspase 3 and 7 activity in P19 cells in response to exogenous BMP2 and ATRA.** P19 cells were treated for 24 hours with vehicle control (0.00001% BSA, 0.001% DMSO, Ctrl), 10 ng/ml recombinant human BMP2 (BMP2), 1 μM ATRA (RA), or 10 ng/ml BMP and 1 μM ATRA (RA+BMP), no treatment (Ctrl) or 70 μM AMA as a positive control. RA acted in concert with BMP2 to increase caspase 3 and 7 activation in P19 cells. Results are mean±s.e.m. (*n*=3). *P<0.05, **P<0.01, ***P<0.001 (Student’s *t*-test).

Combined BMP2 and ATRA treatment has been previously shown to induce apoptosis in P19 cells as indicated by assessment of DNA fragmentation using cell sorting or direct electrophoresis, or by microscopic examination of cells for condensed chromatin (Fujita et al., 1999; Glozak and Rogers, 1996; Glozak and Rogers, 1998). We have been able to corroborate this finding by examining caspase 3 and 7 activation, which are mediators of apoptosis. Treatment of P19 cells with BMP2 and ATRA resulted in a significant increase in the activity of caspase 3 and 7 compared to control, BMP2 or ATRA alone (Fig. 5). To examine whether oxidative stress can have similar effects, P19 cells were treated with the complex III electron transport inhibitor and inducer of oxidative stress, antimycin A (AMA) (García-Ruiz et al., 1995; Turrens et al., 1985). AMA treatment alone also significantly increased caspase activation, supporting the link between ROS and apoptosis.

### Oxidative stress is increased by RA treatment in P19 cells

**Fig. 6.**
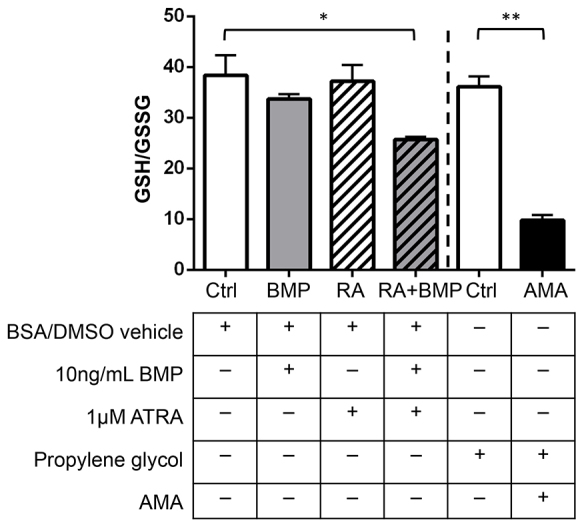
**Quantitation of oxidative stress in P19 cells treated with BMP2 and ATRA.** The ratio of reduced glutathione (GSH) to oxidized glutathione (GSSG) was assayed in P19 cells treated for 16 hours with vehicle control (0.0001% BSA, 0.001% DMSO, Ctrl), 10 ng/ml recombinant human BMP2 (BMP2), 1 μM ATRA or 10 ng/ml BMP combined with 1 μM ATRA (BMP+RA). 70 μM AMA was tested as a positive control and compared to its vehicle, propylene glycol. As expected, treatment of P19 cells with the prooxidant AMA resulted in a significant decrease in GSH:GSSG. In addition, oxidative stress was increased in P19 cells treated with ATRA in combination with BMP2. Results are mean±s.e.m. (*n*=3). *P<0.05, **P<0.01 (Tukey’s test).

The antioxidant GSH provides the main cellular defense against oxidative damage and can be depleted and converted into the oxidized form GSSG in conditions of oxidative stress. Hence, the GSH:GSSG ratio provides a reliable indicator of the oxidative status of a cell. As expected, treatment of P19 cells with a prooxidant AMA resulted in a significant decrease in GSH:GSSG ratio (Fig. 6). Cells that have been treated with BMP2 alone or low dose ATRA alone did not show any significant changes in GSH:GSSG ratio. By contrast, a combined BMP2 and RA treatment resulted in a markedly lower GSH:GSSG ratio. This result indicates that RA along with BMP2 can induce oxidative stress in this cellular model of early embryonic development.

## DISCUSSION

The severity of craniofacial abnormalities can vary widely between individuals, despite similar or identical genetic risk factors and environmental exposures. HPE is a prominent example of such phenotypic variability. Several mechanisms have been proposed to explain this phenomenon, such as interaction of two or more HPE genes in generating the phenotype (Nanni et al., 1999), cumulative effects of mutations in non-classical HPE genes that result in concurrent or sequential partial defects in more than one pathway important for forebrain development (Andersson et al., 2006; Ming et al., 2002), presence of genetic modifiers (Nadeau, 2001), and stochastic and/or epigenetic contributions (Feinberg and Irizarry, 2010), as well as non-linearities in the properties of signaling pathways (Young et al., 2010). The multifactorial etiology of HPE led to the ‘multiple hit’ hypothesis (Ming et al., 2002), in which genetic predisposition puts individuals at risk for manifesting the disease in the presence of other exposures.

This study provides direct evidence for such modifying effects of the environment in a genetic mouse model carrying a predisposing mutation in the *Twsg1* gene. Importantly, even haploinsufficiency sensitized the *Twsg1* mouse embryos to the teratogenic effects of RA, resulting in HPE, similar to *Shh* or *Gli2* haploinsufficiency predisposing to teratogenic effects of prenatal ethanol exposure (Kietzman et al., 2014). To quantify these effects, we used a 3D geometric morphometric analysis of craniofacial shape by microCT (Chong et al., 2012; Johnson et al., 2006; Nagase et al., 2008). We found that the majority of shape variation in *Twsg1^+/−^* mouse embryos with intrauterine exposure to a low dose of ATRA was associated with narrowing of the midface as seen in the human microforms (Roessler et al., 1996), *Noggin*-null mice (Lana-Elola et al., 2011) and in a chick model of HPE (Marcucio et al., 2005; Young et al., 2010). Given that the type of dysmorphology, midfacial narrowing, is similar to that observed in untreated *Twsg1^−/−^* embryos (MacKenzie et al., 2009), we propose that RA treatment moves the phenotypes of heterozygotes towards a mutant phenotype.

The exact underlying mechanisms of this increased sensitivity of TWSG1-deficient embryos to a teratogen like RA are unknown. Given that the only known action of TWSG1 is through binding BMPs in the extracellular space, and we have previously shown that BMP signaling is increased in the absence of TWSG1 (Ross et al., 2001; Sotillo Rodriguez et al., 2009), we speculate that BMP-RA interactions contribute to the enhanced expressivity of HPE in this mouse model. One potential mechanism of this synergy of BMP and RA lies in the observed induction of the BMP pathway members by RA. RA induces *Bmp2* expression, as shown both in this study and in previous research (Heller et al., 1999), and might in other settings also induce *Bmp4* and *Bmp7* (Rodriguez-Leon et al., 1999). The upregulation of *Msx* genes following a BMP and ATRA treatment is particularly interesting because the expression of *Msx2* has been linked to induction of apoptosis in the craniofacial region, including in neural crest cells (Graham et al., 1994) and optic vesicles (Wu et al., 2003).

There is evidence that both BMP and RA pathways cooperate to induce apoptosis *in vivo*, for example during vertebrate limb outgrowth (Rodriguez-Leon et al., 1999) and in *in vitro* systems, including P19 cells, as shown in this and other studies (Glozak and Rogers, 1996; Xi and Yang, 2008). We have previously shown that in *Twsg1^−/−^* embryos increased apoptosis correlates with the degree of severity of craniofacial phenotypes (MacKenzie et al., 2009). Any additional pro-apoptotic factor would be expected to enhance this dysmorphology. In fact, excessive apoptosis is a central common pathway in various craniofacial defects due to exposure to external noxious agents, such as alcohol (Aoto et al., 2008; Sulik, 2005), hypoxia (Smith et al., 2013) or gene mutations (Dixon et al., 2000; Jones et al., 2008; Phelan et al., 1997). This pathological apoptosis appears to be mediated, at least partly, by p53 because genetic or pharmacological inhibition of p53 activation can significantly reduce the frequency of craniofacial defects in *Twsg1^−/−^* and other mouse models (*Tcof*, *Pax3*) of craniofacial and neural defects (Billington et al., 2011; Jones et al., 2008; Pani et al., 2002). Importantly, teratogens such as RA can also induce apoptosis by themselves in craniofacial primordia (Evrard et al., 2000) and activate p53 (Hosako et al., 2007). In this study, treatment of P19 cells with BMP and ATRA also led to a significant upregulation of the p53 target *Trp53inp1*. Similarly, in keratinocytes, RA increases the expression of p53 and proapoptotic caspases and sensitizes the cells to apoptosis by lowering their apoptotic threshold (Mrass et al., 2004). Future studies should address whether BMP and RA pathways can act together to lower the apoptotic threshold during key stages of midline forebrain and facial development *in vivo*.

Another possible intermediate mechanism underlying hyperresponsiveness of embryos to teratogens is oxidative stress. Studies in animal models and humans have implicated ROS generation in the pathogenesis of craniofacial and other birth defects (Chang et al., 2003; Davis et al., 1990; Dong et al., 2008; Kay et al., 2000; Kotch et al., 1995; Loeken, 2004; Ornoy, 2007). Three known environmental causes of HPE, gestational diabetes, fetal alcohol and RA exposure (Cohen and Shiota, 2002), are all associated with elevated levels of ROS (Aoto et al., 2008; Davis et al., 1990; Kay et al., 2000; Ornoy, 2007). In fact, the ability to remove ROS is thought to be a general mechanism to neutralize environmental toxins. One of the reasons why early embryos might be particularly sensitive to free radical damage is their limited antioxidant capability, partly due to an inherent deficiency of the scavengers of ROS, superoxide dismutase and catalase (Davis et al., 1990). Oxidative stress is thought to promote apoptosis, which then disrupts normal development. Supplementation with exogenous antioxidants, including *N*-acetylcysteine, vitamin C, vitamin E, superoxide dismutase or catalase, in animal models has produced promising results in terms of reducing apoptosis and dysmorphology (Aoto et al., 2008; Kotch et al., 1995; Loeken, 2004; Simán and Eriksson, 1997; Wentzel and Eriksson, 1998). The current study shows that ATRA in combination with BMP can lower GSH to GSSG ratios, indicating induction of oxidative stress in P19 cells. It should also be noted that other cellular processes might also be disrupted by RA and contribute to the phenotypic heterogeneity in *Twsg1* mutant embryos, such as premature differentiation induced by increased levels of RA (Laue et al., 2011).

RA (an analog of vitamin A) has also been proven to cause birth defects in humans, including central nervous system abnormalities such as HPE (Cohen and Shiota, 2002). The association between vitamin A and birth defects comes from studies in which high doses were used. For example, in a study of 154 human pregnancies, *in utero* exposure to isotretinoin (prescribed to treat severe cystic acne) was associated with a high risk of congenital malformations (relative risk 25.6) (Lammer et al., 1985). All women took oral isotretinoin at some point during the first 10 weeks after conception. This has led to an increased awareness about teratogenic effects of retinoic acid and reduced exposure, although not complete elimination as it continues to be prescribed for the treatment of acne, sun-damaged skin, psoriasis, prevention of nonmelanoma skin cancer, and for cancer chemotherapy (Mrass et al., 2004). Vitamin A is classified in the U.S. Food and Drug Administration’s Pregnancy Category X; therefore, doses that exceed the recommended daily allowance (RDA) should be avoided by women who are or might become pregnant (Office of Dietary Supplements National Institutes of Health: Dietary supplement fact sheet: vitamin A and carotenoids, 2006; http://ods.od.nih.gov/factsheets/VitaminA-HealthProfessional/). However, vitamin A, when used in recommended doses, is needed for normal fetal growth (van den Broek et al., 2010) and is safe in the general population (Dudas and Czeizel, 2002).

In summary, TWSG1-deficient mice represent a genetic mouse model of a mutation with low penetrance that sensitizes embryos to environmental influences. The mechanisms underlying these gene-environment interactions are poorly understood. Given that similar biological processes appear to be involved in the pathogenesis of a variety of birth defects, and in response to various teratogens, better understanding of these interactions will likely be applicable to other birth defects beyond craniofacial malformation.

## MATERIALS AND METHODS

### Mice

Mice with a targeted mutation in *Twsg1* (*Twsg1^tm1.1 Mboc^*) were as described previously (Petryk et al., 2004). Wild-type (WT) mice were purchased from Jackson Laboratories. All mice were of the C57BL/6 background. Presence of a spermatic plug was counted as embryonic day 0.5 (E0.5). Pregnant females were treated by gavage with all-trans retinoic acid (ATRA, Sigma, St Louis, MO) in corn oil at doses of 3.75 or 7.5 mg/kg of body weight (Kotch et al., 1995) on the morning (10 am) of E7.5, which is a well defined teratogenic window for the induction of HPE (Higashiyama et al., 2007; Lipinski et al., 2010). Subsequently the pregnant females were killed by CO_2_ inhalation, embryos were isolated at E9.5 or E10.5, and assessed for external phenotypes under the dissecting microscope, including telencephalic vesicle abnormalities consistent with HPE and neural tube defects. For geometric morphometric shape analysis, embryos were collected at E11.5 and fixed in 4% paraformaldehyde with glutaraldehyde (Schmidt et al., 2010). Mice were housed in specific pathogen-free (SPF) conditions. Standard chow and water were provided *ad libitum*. All animal procedures were approved by the University of Minnesota Institutional Animal Care and Use Committee.

### Geometric morphometric shape analysis

GM analysis of craniofacial shape was performed by microCT. Embryos were scanned at 5 μm resolution with a Scanco μCT 35 Scanner (Scanco Medical, Brüttisellen, Switzerland). A detailed description of this technique and computation methods have been previously published (Chong et al., 2012; Young et al., 2010; Young et al., 2007). A set of 45 landmarks were used to define the morphology of the embryonic face and forebrain using established protocols (Boughner et al., 2008; Parsons et al., 2011; Schmidt et al., 2010). Landmark data are then aligned using a generalized least-squares Procrustes superimposition algorithm to remove size, and place all individuals into a common shape space (Mitteroecker and Gunz, 2009). A series of linear combinations of variables was created [principal components (PCs)] that explained successively smaller proportions of total variance. PC1 is computed to capture the largest proportion of variation in the original measurements.

### Cell culture and treatments

P19 mouse embryonic carcinoma cells (ATCC CRL-1825) (Glozak and Rogers, 1996) were cultured in minimal essential medium (MEM) with 10% fetal bovine serum and antibiotics, maintained by splitting 10-fold every 2 days. Cells were treated with recombinant human BMP2 (R&D Systems, Minneapolis, MN) and/or all-trans retinoic acid (ATRA). BMP2 was dissolved as a stock at 100 ng/μl in 4 mM HCl, 0.1% BSA. ATRA was dissolved in DMSO and kept as a stock at 10^−2^ M. All cells in BMP and ATRA treatment experiments were adjusted with non-solute-containing vehicles to final concentrations of 0.0001% (w/v) BSA and 0.01% (v/v) DMSO. Antimycin-A (AMA, Sigma, St Louis, MO) was prepared in propylene glycol at 6 mg/ml.

### Gene expression

P19 cells were plated into six-well plates with 20,000 cells per well and allowed to grow overnight. Medium was removed and replaced with treatment media containing test compounds. For RA induction experiments, cells were treated either with 1 μM ATRA or with DMSO vehicle in the same v/v dilution. For BMP and RA experiments, cells were treated with vehicle control, 10 ng/ml BMP2 with DMSO ATRA vehicle control, 1 μM ATRA with BSA BMP2 vehicle control, or BMP2 combined with ATRA. In both sets of experiments, media were removed after 24 hours and 1 ml of Trizol (Invitrogen, Carlsbad, CA) was added for RNA isolation according to manufacturer’s instructions. cDNA was prepared from RNA samples by reverse transcription using the Thermoscript reverse transcription sysetem (Invitrogen, Carlsbad, CA, USA). Quantitative PCR (qPCR) was performed using 2× SYBR green mastermix with ROX from SABiosciences on an MX3000p thermocycler (Stratagene/Agilent Technologies, La Jolla, CA, USA) and analyzed using the MxPro software (Stratagene). Primers used for the following genes *Bmp2*, *Crbp1*, *Cyp26a1*, *Gapdh*, *Hoxa1*, *Hoxb1*, *Msx1*, *Msx2*, *Rara*, *Rarb* and *Trp53inp1* are shown in supplementary material Table S1.

### Caspase 3 and 7 activity assay

Caspase 3 and 7 activity was measured using reagents from the Apotox-glo triplex assay kit (Promega, Madison, WI) according to the manufacturer’s protocol. Briefly, cells were plated in a white-walled clear-bottomed 96-well plate with 2500 cells per well, then test compounds were added, diluted in growth medium. Cells were treated with vehicle control, 10 ng/ml BMP2 with DMSO ATRA vehicle control, 1 μM ATRA with BSA BMP2 vehicle control, or BMP2 combined with ATRA. After 24 hours, caspase activation was measured by addition of luminogenic caspase substrate and measured in a Centro XS^3^ LB 960 Microplate Luminometer (Berthold Technologies, Bad Wildbad, Germany).

### Glutathione ratio assay

P19 cells were assayed for ratio of reduced to oxidized glutathione using the GSH/GSSG-glo kit (Promega) according to the manufacturer’s instructions. Briefly, cells were plated in a white-walled clear-bottomed 96-well plate with 2500 cells per well, then test compounds were added, diluted in growth medium. Cells were treated with BMP and RA vehicle control, 10 ng/ml BMP2 with DMSO ATRA vehicle control, 1 μM ATRA with BSA BMP2 vehicle control, BMP2 combined with ATRA, propylene glycol AMA vehicle control or 70μM AMA. After 16 hours cells were washed once with HBSS then lysed and analyzed for GSH and GSSG content using kit-provided reagents for luminogenic reactions.

### Statistical analysis

Chi-squared tests, Student’s *t*-tests and ANOVA combined with Tukey’s multiple comparison tests were performed using Prism4 (GraphPad Software, San Diego, CA). Significance was accepted at *P*<0.05. Fisher’s exact test was used to compare frequencies of embryonic phenotypes between different genotypes and with and without RA treatments.

## Supplementary Material

Supplementary Material
