# Supplementary material for: Impact of retinoic acid exposure on midfacial shape variation and manifestation of holoprosencephaly in Twsg1 mutant mice
Source: Dis Model Mech. 2014 Dec 2;8(2):139–46. doi: 10.1242/dmm.018275 (PMC4314779; doi:10.1242/dmm.018275)
Supplement: Supplementary Material [file supp_8_2_139__index.html]

Impact of retinoic acid exposure on midfacial shape variation and manifestation of holoprosencephaly in Twsg1 mutant mice — Supplementary Material 

# Impact of retinoic acid exposure on midfacial shape variation and manifestation of holoprosencephaly in *Twsg1* mutant mice

## DMM018275 Supplementary Material

**Files in this Data Supplement:**

- **Supplementary Material**
