# Supplementary material for: Impact of retinoic acid exposure on midfacial shape variation and manifestation of holoprosencephaly in Twsg1 mutant mice
Source: Dis Model Mech. 2014 Dec 2;8(2):139–46. doi: 10.1242/dmm.018275 (PMC4314779; doi:10.1242/dmm.018275)
Supplement: Supplementary Material [file supp_8.2.139_DMM018275.pdf]

Supplemental Table 1. Primers used in qPCR

Gene	Forward 5' to 3'	Reverse 5' to 3'
Bmp2	tggaagtggcccatttagag	tgacgcttttctcgtttgtg
Crbp1	atccgcacgctgagcacttttc	cactggagtttgtcaccatccc
Cyp26a1	tccaacctgcacgattcctc	agccactgctccagacaactg
Gapdh	tgcaccaccaactgcttag	gatgcagggatgatgttc
Hoxa1	ccaaaacagggaaagttgga	gcgctcgtgtaaggtacttgt
Hoxb1	gctccaacaaaactcggggta	cgacggatgaaaatagcttcca
Msx1	ccaggtgaagatctggttcc	caggaaaagagaggccgaag
Msx2	cctcaggaaacacaagacca	agttgatagggaagggcaga
Rara	ctgtaagggcttcttccg	agtcttaatgatgcactt
Rarb	tggatgttctgtcagtgagtccc	cgatggattgagcagtatgccg
Trp53inp1	aagtggtcccagaatggaagc	ggcgaaaactcttgggttgt
